# Investigation of the fungiform papillae number in children with tooth number anomalies

**DOI:** 10.1007/s00784-024-05696-1

**Published:** 2024-05-03

**Authors:** Belgin Alp, Elif Ece Kalaoglu, Ali Mentes

**Affiliations:** 1 Trabzon Dental Public Health Hospital, Trabzon, Turkey; 2 Department of Pediatric Dentistry, Faculty of Dentistry, İstanbul Gelişim University, Istanbul, Turkey; 3 Department of Pediatric Dentistry, Faculty of Dentistry, Marmara University, Istanbul, Turkey

**Keywords:** Hyperdontia, Hypodontia, Fungiform papillae, Tooth number anomaly

## Abstract

**Objective:**

This cross-sectional study investigated the association between fungiform papillae (FP) numbers and tooth number anomalies in children, considering variables related to hypodontia and hyperdontia. The aim was to explore this association while adjusting for age and sex differences.

**Materials and methods:**

A total of 144 children (aged 8–10) were categorized into hypodontia (*n* = 48), hyperdontia (*n* = 48), and control groups (*n* = 48). Clinical and radiographic diagnoses were used to classify tooth number anomalies. Hypodontia was categorized by number and location, while hyperdontia was categorized by number, shape, and location. FP were assessed using the Denver Papillae Protocol. Data analyses were performed using NCSS software, with *p* < 0.05 considered statistically significant.

**Results:**

The hypodontia group (22.5 ± 8.4) exhibited significantly lower FP than the control group (30.4 ± 9.2) and the hyperdontia group (27.9 ± 7.8) (*p* < 0.0005, *p* = 0.003, respectively). No significant difference existed between the hyperdontia and control groups. FP numbers in hypodontia subgroups showed no significant differences based on teeth agenesis numbers or locations. Similarly, hyperdontia subgroup analyses revealed no significant differences in FP numbers based on supernumerary teeth shapes (supplemental, conical, tuberculoid, paramolar) or the numbers of supernumerary teeth.

**Conclusions:**

The lower FP numbers in children with hypodontia suggested an association between teeth and FP number. However, the non-significant difference in FP numbers with hyperdontia underscored the complexity of tooth development, warranting further investigations.

**Clinical relevance:**

Children with hypodontia may exhibit distinct FP numbers compared to those without tooth number anomalies.

**Supplementary Information:**

The online version contains supplementary material available at 10.1007/s00784-024-05696-1.

## Introduction

Tooth development is a sophisticated process driven by intricate interactions between the oral epithelium and the ectomesenchyme. The oral epithelium originates from the ectoderm, and the ectomesenchyme is derived from the neural crest [1]. This process involves a complex interplay of signalling centers, transcription factors, and pathways [1, 2]. Notably, teeth and taste buds, being neurosensory organs, share a common developmental origin rooted in the interactions of epithelial and progenitor cells [3]. The development of structures like teeth, hair, glands, intestinal villi, and fungiform papillae (FP), vital for taste perception [4], involves analogous epithelial-mesenchymal interactions [5–8]. Crucial signaling pathways are engaged in the formation of teeth and tongue papillae [9, 10]. Animal studies have revealed shared genes influencing the development of both taste buds and teeth. For instance, Bloomquist et al. [3] linked tooth and taste bud density through Wingless signals, also identifying signals that distinguish organ identity from a common epithelial lamina using BMP and Hedgehog signaling. Recognizing these developmental parallels between teeth and taste papillae offers insights into the etiology of dental anomalies, including hypodontia.

Hypodontia, characterized by the reduction in tooth number due to the absence of one or more tooth germs, stands as one of the most common developmental anomalies [11, 12]. Its prevalence ranges from 3 to 10%, depending on the study population. It predominantly affects the permanent dentition, excluding third molars, and shows a slight female-to-male predilection of about one-and-a-half to one [11]. In the Caucasian population, Soxman [11] reported that 80% of individuals with hypodontia exhibited only one or two agenesis. In Turkey, the prevalence of hypodontia in the permanent dentition was reported to be 6.02% [13], similar to that found in the Slovenian population, which is considered part of Central Europe, where a prevalence of 6.9% was reported [14].

Hyperdontia, characterized by an excess number of teeth in the dentition, exhibits ethnic variations, with a prevalence ranging from 0.1% to 3.8% in Caucasians and slightly higher rates in Asian populations [15]. In the Turkish population, the prevalence of hyperdontia was found to be 0.5% [16]. Males show a two-to-one predominance over females in cases of hyperdontia. Single-tooth hyperdontia is reported in approximately 80% of cases, primarily observed in the permanent dentition, with about 95% of these cases affecting the maxillary arch, particularly in the anterior region [11].

Previous studies have explored the relationship between the number of FP and various factors such as taste perception, taste sensitivity, body mass index, age, gender, different habits, and diseases [4, 17–20]. However, to date, no study has investigated the relationship between the FP number and orodental abnormalities in humans. This study aimed to investigate the association between the FP number in children with hypodontia or hyperdontia. The null hypotheses were as follows: 1) There would be no significant difference in the FP number between the control and hypodontia groups or between the control and hyperdontia groups. 2) There would be no association between the FP number and variables related to teeth agenesis, including their quantity and location. 3) There would be no association between the FP number and variables related to supernumerary teeth, including their quantity, location, and shapes.

## Material and methods

### Subjects

We recruited healthy children who presented to the Department of Pediatric Dentistry at Marmara University Faculty of Dentistry, Istanbul, Turkey for dental and radiographic examinations. The study was conducted in accordance with Declaration of Helsinki and was approved by Marmara University Clinical Research Ethics Committee (protocol #2016–62). The parents of each participant signed the volunteer information & consent forms.

Subjects were required to meet specific criteria outlined in the inclusion criteria:

- Healthy children with no systemic diseases.
- Radiographs confirming the presence of hypodontia or hyperdontia.
- Age between 8 and 10 years.

Exclusion Criteria:

- Recent history of drug use associated with infections or surgeries within the last month.
- Patients exhibiting a severe gagging reflex or those who experienced issues during tongue drying and dyeing.
- History of premolar extraction according to the parents in the related region.

A control group of healthy children aged between 8 and 10 years, consisting of an equal distribution of 50% girls and 50% boys, without any tooth number anomalies, was selected for comparison.

### Sample size calculation

The sample size calculation was conducted using the online Power calculator for binary outcome equivalence trial available at https://www.sealedenvelope.com/power/binary-equivalence/. The calculation was based on specific parameters: an alpha level of 0.05, a power of 80% (1-beta), a success rate of 90% in both groups, and an equivalence limit of 18%. These parameters were chosen to control both Type I and Type II errors effectively. The calculated sample size indicated that a total of 144 patients were required, with 48 individuals assigned to each group.

### Study design

The medical and dental histories of the participants were recorded along with their demographic data (name, gender, place of birth, date of birth). Subsequently, visual dental and radiological examinations were conducted for each child. Comprehensive records were maintained for each child, documenting the specific details of hypodontia (number and location of missing teeth) and hyperdontia (number, location, and shape of supernumerary teeth). The tongues were stained, post-procedure photographs were taken, and then FP were counted following the Denver Papillae Protocol [21]. The study was conducted from March 2016 to October 2017, and the data were carefully labelled, registered, and analysed. All patients underwent a re-evaluation in the subsequent year, involving the examination of the new panoramic radiographs to assess treatment effectiveness and provide necessary follow-up every 6 months.

### Identification of hypodontia/ hyperdontia

In cases of missing teeth, the patients' dental histories were meticulously investigated with input from their parents. To diagnose tooth agenesis, panoramic radiographs were taken for all patients, and periapical radiographs were selectively used in cases with artifacts or image quality concerns in the panoramic radiographs. This combined approach allowed us to record the number and location of any missing teeth in the anterior or posterior regions of the upper and lower jaws while adhering to the As Low As Reasonably Achievable (ALARA) principle. Teeth agenesis were classified by location as anterior, posterior and anterior + posterior [11]. Hypodontia group was also categorized into three subgroups: 1 to 2 teeth agenesis; 3 to 5 teeth agenesis; and more than 6 teeth agenesis.

We diagnosed clinically erupted supernumerary teeth during the oral examination. The unerupted supernumerary teeth were detected radiographically. The surgical treatment involved the removal of unerupted supernumerary teeth and/or bonding an attachment to the underneath permanent tooth. The surgical procedure was performed either locally or under general anesthesia. Cone-beam computed tomography (CBCT) images were obtained when panoramic or periapical X-rays could not adequately display the precise location of the supernumerary teeth. We recorded tooth types according to their morphologies as supplemental or rudimentary. Rudimentary teeth were classified as conical (peg-shaped), tuberculoid (barrel-shaped) in the anterior with one or more cusps, and paramolar (small premolar or molar-like in shape/molariform) [11]. Hyperdontia group was also categorized into three subgroups: one supernumerary tooth; double supernumerary teeth; and more than two supernumerary teeth.

### Obtaining the images of tongue and determination of FP numbers

**Fig. 1:**
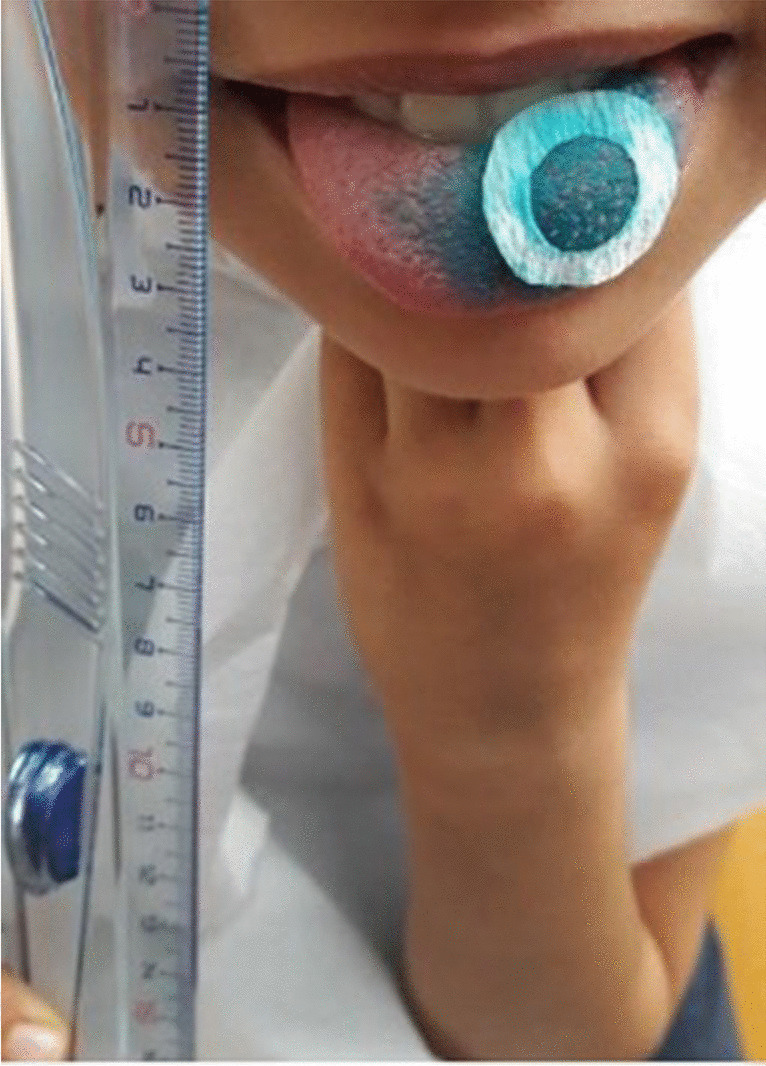
The patient's position for tongue photo

The Denver Papillae Protocol was used to take pictures of the tongue and determine the FP number. The researchers showed the children an educational video in which they painted each other's tongue and took photographs. The researcher (BA) demonstrated how to pose for the photographs. Before painting the tongue, the researchers ensured that the children could place their hands under their chin in an appropriate position and keep their tongue stabilized (Fig. 1). The researcher (BA) dried the tongue with a paper towel and applied one or two drops of 1/36 diluted blue food dye (Brilliant Blue FCF, Sensient Technologies Europe GmbH, Geesthacht, Germany). A circle with a diameter of 10 mm was cut from filter paper and placed on the left side of the midline of the tongue. The children were instructed to stick out their tongue and hold it between their teeth. Then, photographs were taken using a digital compact camera (Nikon COOLPIX P610, Tokyo, Japan). As Denver papillae protocol suggested, training was performed via https://www.jove.com/v/52860/denver-papillae-protocol-for-objective-analysis-of-fungiform-papillae. The researchers (BA, EEK) independently scored each photograph using ImageJ software (version 1.46r, 32-bit). ImageJ facilitates the display, editing, analysis, processing, storage, and printing of images in various bit depths, including 8-bit, 16-bit, and 32-bit. During the scoring process, if the difference between the two FP raw scores exceeded 10%, both scored photos were reviewed together. If consensus was still not reached, a third researcher's opinion (AM) was sought to resolve discrepancies [21].

### Statistical evaluation

The Kolmogorov–Smirnov test was used to determine whether continuous variables were normally distributed. Descriptive statistics were presented as frequencies and percentages for the sex variable and mean and standard deviation for the age and FP number variable. The association between the study groups (hypodontia, hyperdontia, control) and sex was evaluated using the chi-square test. The association between the study groups and age was also evaluated using the one-way analysis of variance (ANOVA) test followed by Tukey's multiple comparison test for subgroup analysis. Multivariate linear regression model was fitted for the FP number variable. This model included: study groups (type), sex, and age. Data analysis was performed using NCSS (Number Cruncher Statistical System) 2007 Statistical Software (Utah, USA). The significance level was set at *p* < 0.05.

## Results

Initially, 156 children (52 per group) were screened for eligibility. Among them, four patients in the hypodontia group developed teeth in the years 2017 and 2018, resulting in a reduction of the hypodontia group to 48 children. To maintain uniformity, eight children (2 boys and 2 girls from each group) were randomly excluded from both the hyperdontia and control groups (*n* = 8). This resulted in 144 children confirmed as eligible and included in the study, comprising 48 with hypodontia (18 boys, 30 girls), 48 with hyperdontia (31 boys, 17 girls), and 48 without any tooth number anomalies (24 boys, 24 girls). The intraclass correlation coefficient for FP number measurements demonstrated high consistency among researchers within specific group; control group, hypodontia group, hyperdontia group, and the entire study cohort (0.99, 95% CI: 0.98–0.99).

FP counts were based on the data collected by one observer (BA) to ensure consistency. Examination of participant demographics revealed no significant age differences between groups (Table 1). The mean FP number for boys was 26.9 ± 8.5, and for girls, it was 25.9 ± 9.6. Importantly, there was no statistically significant difference in mean FP numbers between boys and girls (*p* = 0.46). In terms of FP distribution, counts ranged from 9 to 55 papillae within a 0.75 cm^2^ area, with an overall mean of 26.9 ± 9.1 (Table 2). The effects of age and sex parameters on FP numbers were not significant (*p* > 0.05) whereas hypodontia, hyperdontia and control groups were significant (*p* = 0.001) when using multivariate linear regression analysis (Table 2).

**Table 1:** Distribution of control, hypodontia and hyperdontia groups by age and sex

		Control		Hypodontia		Hyperdontia		*p*
Age		8.8 ± 0.8		9.3 ± 0.8		9.1 ± 0.9		0.114^**1**^
Sex	Male	24	50%	18	37.5%	31	64.6%	**0.029**^**2**^
	Female	24	50%	30	62.5%	17	35.4%	

^1^One Way Analysis of Variance

^2^ Chi Square test

**Table 2.**
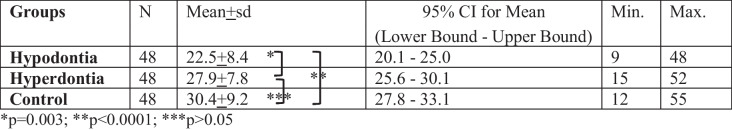
Mean, standard deviation, upper and lower bounds within 95% confidence interval, minimum, maximum values of FP number, and multiple comparison by groups

*p=0.003; **p<0.0001; ***p>0.05

Note: Multivariate linear regression analysis revealed that the effects of age and gender on FP number were not statistically significant (*p* = 0.837, *p* = 0.452 respectively). However, the ‘groups’ variable exhibited a significant impact (Coefficient = 3.0, SE = 0.9, Beta = 0.3, t = 3.4) indicating a statistically significant association with FP number (*p* = 0.001)

The mean FP number in the hypodontia group was found to be 22.5 ± 8.4, while in the hyperdontia group, it was 27.9 ± 7.8. In the control group, the mean FP number was 30.4 ± 9.2 (Table 2). A statistically significant difference was observed among the groups (*p* < 0.0005). Specifically, the mean FP number in the hypodontia group was significantly lower than that in both the control group (*p* < 0.0001) and the hyperdontia group (*p* = 0.003). However, there was no statistically significant difference in the mean FP numbers between the control and hyperdontia groups (*p* > 0.05) (Table 2).

### Hypodontia group evaluation

Among the 48 children with hypodontia, a total of 186 cases of tooth agenesis were observed. Upon examining the distribution of tooth agenesis by region, it was found that 42% (*n* = 22) occurred in the anterior region, 38% (*n* = 16) were limited to the posterior region, and 20% (*n* = 10) affected both the anterior and posterior regions. The mean number of FP in the anterior, posterior, and anterior + posterior subgroups were 23.5 ± 8.4, 23.9 ± 8.6, and 20.1 ± 7.8, respectively (SI Table 1). Statistical analysis revealed no significant difference in the teeth agenesis number among the anterior, posterior, and anterior + posterior subgroups in the hypodontia group (*p* = 0.47) (SI Table 1). However, it is worth noting that patients with tooth agenesis in both the anterior and posterior regions had the fewest missing teeth.

**Fig. 2:**
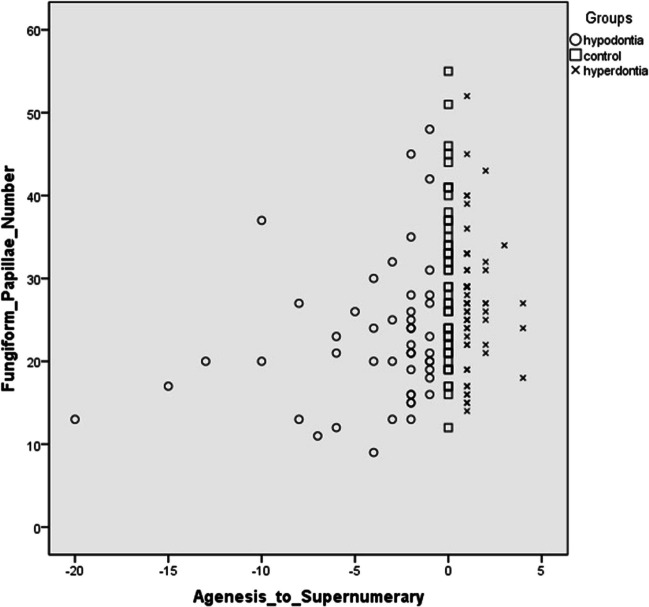
Distribution of FP numbers according to tooth number anomalies. Agenesis_to_Supernumerary scale; 0 represented control group meaning no supernumerary teeth or tooth agenesis in children. Lower than 0 down to -20 showed the number of tooth agenesis in children. Higher than 0 up to 5 showed the number of supernumerary teeth in children. Fungiform_Papillae_Number scale; 0 up to 60 represented the fungiform papillae number within a circle with a diameter of 10 mm

The mean FP number for subgroups with 1 to 2 teeth agenesis, 3 to 5 teeth agenesis, and more than 6 teeth agenesis were 24.5 ± 8.5, 22.1 ± 7.5, and 19.5 ± 7.7, respectively (SI Table 2). These findings suggested a pattern of decreasing FP numbers with an increasing number of tooth agenesis (Fig. 2). However, it's important to note that our analysis did not reveal statistical significance.

### Hyperdontia group evaluation

A total of 70 supernumerary teeth were identified in the hyperdontia group. Among them, 10% (*n* = 7) were supplemental, 45% (*n* = 32) were conical, 28% (*n* = 20) were tuberculoid, and 14% (*n* = 11) were paramolar teeth. When examining the mean FP number according to the tooth types in the hyperdontia group, the highest FP number was observed in patients with paramolar teeth (30.3 ± 8.6), followed by conical teeth (28.2 ± 8.3), supplemental teeth (26.2 ± 9.1), and tuberculoid teeth (24.8 ± 6.9). However, there were no significant differences between the subgroups (*p* = 0.46) (SI Table 3).

In the hyperdontia group, 73% of the patients had one supernumerary tooth (*n* = 35), 18% had double supernumerary teeth (*n* = 9), and 8% had more than two supernumerary teeth (*n* = 4). The mean number of FP in these subgroups was 27 ± 8.4, 28.2 ± 6.6, and 25.8 ± 6.7, respectively (Fig. 2) (SI Table 4). There was no significant difference or relationship observed between the number of supernumerary teeth and the FP number (*p* = 0.86) (SI Table 4).

## Discussion

We conducted this study to explore the relationship between the number of FP and tooth anomalies in humans. Drawing inspiration from Bloomquist et al. [3], which focused on cichlid fish, our study is among the first to investigate this association in humans. On the other hand, Wong et al. [22] investigated the effects of tooth agenesis on bitter taste perception, our research specifically delves into the association between the number of teeth and FP in humans.

Various methods have been used to determine the number of FP. Miller et al. [23] initially counted papillae on human cadaver tongues. Subsequent studies in live humans involved painting the tongue with food dye and capturing tongue images with a video-microscope [24–27]. However, this method proved time-consuming, and a digital camera was later employed as a more efficient alternative [27]. Previous studies have developed an algorithm to count FP in tongue images, replacing the traditional manual counting method [28, 29]. Various computer systems or software are necessary for determining FP count; further research is needed to confirm their accuracy.

The Denver Papilla Protocol, developed by Nuessle et al. [21], is a currently popular method for FP counting as it requires less time, tolerates tongue and head movement better, and is cost-effective. Another current method for FP counting involves the use of a confocal laser scanning microscope, which can also determine the number of taste buds, but it is costly and requires expert knowledge [30]. In a study similar to ours, a group of children aged 8–11 years were painted with blue food dye and photographed with a digital camera to determine FP count [31]. Similarly, a study on 101 children aged 11–15 years also used blue food dye and investigated the relationship between FP count and taste sensitivity [32]. Other recent studies have similarly utilized the Denver Papilla Protocol method to assess FP count [33–35]. We excluded two individuals with a recent history of medication use within the last month, as such factors can potentially influence FP morphology, as evidenced by Piochi et al. [36]. We experienced difficulties while taking tongue photos from these young children. Three children with severe gagging reflex and two children with inadequate tongue drying and dyeing were dismissed. Additionally, one patient with a reported history of unknown extraction in the relevant premolar region, as indicated by the parents, was excluded to eliminate any bias associated with hypodontia.

The relationship between FP number and age has been a subject of investigation in various cross-sectional studies. For instance, Correa et al. [37] examined changes in FP density across different age groups, including children aged 7–8 years, 9–10 years, 11–12 years, and adults aged 20–24 years. While they found no statistically significant difference in the number of FP between children in the 7–8 years age group and those in other age groups, they observed that children in this particular age group had a higher number of FP compared to adults. Another study on 83 children aged 8–11 years showed a negative correlation between age and the number of FP, indicating a decrease with age [31]. In our previous cross-sectional study involving children aged 5–10 years, a decrease in FP numbers with age was reported [33]. This observation suggested a potential association between FP number and age within the specific age range. Therefore, our present study targeted the narrow age range of 8–10 years to minimize the potential confounding effect of age on FP number. Remarkably, a study involving a wide age range (21–84) showed that with every 5-year increase in age groups, the mean density of FP decreased by 2.8 papillae/cm^2^ [38]. However, it is noteworthy that studies in adult populations have reported mixed results concerning the relationship between FP number and age, with some finding no significant differences. For instance, Bajec and Pickering [39] observed no significant differences in FP number across age groups in adults. Similarly, Feeney and Hayes [40] and Masi et al. [41] found no significant associations between age and FP number in their studies.

Numerous cross-sectional studies have explored the association between sex and the quantity of FP. In our study, we investigated this relationship and found no statistically significant difference in the number of FP between males and females, consistent with previous studies [31, 37, 39, 41]. Jilani et al. [31] reported similar findings, with the range between 14 and 46 in a 6 mm diameter circle. Additionally, Sobek and Jagielski [32] found no significant disparity in FP numbers between boys and girls in children aged 11–15 years, with a median of 27 and a range from 12 to 47. Correa et al. [37] and Fogel and Blissett [42] also reported no significant sex difference in FP numbers in studies with children. Fogel and Blissett's study [42], for instance, found that the mean number of FP counted was 37.3 ± 9.9 with a density ranging between 23–67/cm^2^. Kalaoglu et al. [33] found that girls had higher FP numbers than boys aged 5–10 years. On the other hand, studies involving larger adult populations have produced differing results. Fischer et al. [38] reported that females had more FP than males in a study with 2371 adult participants. These contrasting findings suggest that the relationship between FP numbers and sex may be influenced by various factors such as age, ethnicity, and environmental conditions [36–38]. Further research is needed to better understand the FP numbers, considering various factors that may contribute to the observed differences.

The primary limitation of this cross-sectional study design lies in its simultaneous assessment of FP number and tooth number anomalies, as it does not provide evidence of a temporal relationship between exposure and outcome. Since FP number can change over time, a deeper understanding would require a longitudinal study. Future research could greatly benefit from repeated assessments over time and expansion to different age groups, although challenges in assessing the papillae of younger children should be considered. Despite these limitations, we believe this study is crucial as the first investigation on this subject, contributing to the detection of a relationship between fungiform papilla number and tooth number.

This research was centered around comparing two different tissues with similar phenotypes and developmental delays. The significance of this study lies in the observation that the FP numbers is reduced in cases of hypodontia. The hyperactivity of dental lamina is the most acceptable and widely accepted theory for supernumerary teeth. Lu et al. [43] mentioned that it is unclear whether the hyperactivity of the dental lamina, which leads to hyperdontia, particularly in non-syndromic cases, has a genetic or environmental origin. Our study did not identify any significance in cases of hyperdontia, indicating the need for more comprehensive investigations on this subject. Future investigations could explore the specific mechanisms linking tooth development anomalies and taste bud alterations and guiding personalized approaches for individuals with dental anomalies.

In conclusion, our study revealed a statistically significant reduction in the average number of FP in the hypodontia group compared to both the hyperdontia and control groups, in line with our initial hypotheses. However, the absence of a statistically significant difference between the control and hyperdontia groups underscored the intricate relationships between tooth and papillae numbers, prompting further investigation. The null hypotheses concerning the quantity, location, and shape of tooth number anomalies were accepted, as no associations were discovered between the FP number and variables associated with teeth agenesis, or related to supernumerary teeth. These findings not only emphasized the complexity of these relationships but also served as a launching point for future investigations.

### Supplementary Information

Below is the link to the electronic supplementary material.

Supplementary file1 (DOCX 15.8 KB)

## Data Availability

The entirety of the data analyzed in this study has been included in the published article. Upon making a reasonable request, the corresponding author will provide access to the raw data.
