# Supplementary material for: Investigation of the fungiform papillae number in children with tooth number anomalies
Source: Clin Oral Investig. 2024 May 3;28(5):297. doi: 10.1007/s00784-024-05696-1 (PMC11068673; doi:10.1007/s00784-024-05696-1)
Supplement: Supplementary file 1 — Supplementary file1 (DOCX 15.8 KB) [file 784_2024_5696_MOESM1_ESM.docx]

**Supplementary Information (SI)**

**SI Table 1.** The mean number of fungiform papillae according to the teeth agenesis locations

| **Location** | **n** | **Fungiform papillae number** |
| --- | --- | --- |
| Anterior | 22 | 23.5 ± 8.4 |
| Posterior | 16 | 23.9 ± 8.6 |
| Anterior+posterior | 10 | 20.1 ± 7.8 |
|  |  | p=0.47 |

One-Way Variance Analysis

**SI Table 2.** The mean number of fungiform papillae according to the number of teeth agenesis

| **Teeth agenesis** | **n** | **Fungiform papillae number** |
| --- | --- | --- |
| 1-2 | 22 | 24.5 ± 8.5 |
| 3-5 | 16 | 22.1 ± 7.5 |
| >6 | 10 | 19.5 ± 7.7 |
|  |  | p=0.22 |

One-Way Variance Analysis

**SI Table 3.** The mean number of fungiform papillae according to supernumerary tooth types

| **Tooth types** | **n** | **Fungiform papillae number** |
| --- | --- | --- |
| Supplemental | 4 | 26.2 ± 9.1 |
| Conical | 26 | 28.2 ± 8.3 |
| Tuberculoid | 15 | 24.8 ± 6.9 |
| Paramolar | 3 | 30.3 ± 8.6 |
|  |  | p=0.46 |

One-Way Variance Analysis

**SI Table 4.** The mean number of fungiform papillae according to the number of supernumerary teeth

| **Number of supernumerary teeth** | **n** | **Fungiform papillae number** |
| --- | --- | --- |
| 1 | 35 | 27 ± 8.4 |
| 2 | 9 | 28.2 ± 6.6 |
| >2 | 4 | 25.8 ± 6.7 |
|  |  | p=0.86 |

One-Way Variance Analysis
